# Usefulness of Bcl-2 Expression and the Expression of Cytoplasmic Immunoglobulin Light Chains in the Differentiation Between B-Cell Lymphoma and Reactive Lymphocytic Proliferations in FNA

**DOI:** 10.3390/ijms20112648

**Published:** 2019-05-29

**Authors:** Andreja Brozic, Ziva Pohar Marinsek, Simon Bucek, Maja Cemazar, Veronika Kloboves Prevodnik

**Affiliations:** 1 Department of Cytopathology, Institute of Oncology, 1000 Ljubljana, Slovenia; abrozic@onko-i.si (A.B.); ZPohar@onko-i.si (Z.P.M.); sbucek@onko-i.si (S.B.); 2 Department of Experimental Oncology, Institute of Oncology, 1000 Ljubljana, Slovenia; MCemazar@onko-i.si

**Keywords:** B-cell lymphoma, flow cytometry, inconclusive surface immunoglobulin light chains, Bcl-2, cytoplasmic immunoglobulin light chains, fine needle aspiration

## Abstract

Flow cytometry is helpful in differentiating between B-cell lymphoma (BCL) and reactive lymphocytic proliferation (RLP) in FNA biopsies. However; the presence of inconclusive surface immunoglobulin light chains (sIg LC) poses a problem. We investigated the usefulness of additional tests; namely Bcl-2 expression and expression of cytoplasmic Ig LC (cIg LC), mainly on samples with inconclusive sIg LC. Both tests were performed on 232 FNA samples from lymph nodes. Bcl-2 alone was determined qualitatively and quantitatively on 315 samples. The quantitative test was correctly positive in 76% of cases and falsely negative in 24%. The correctly positive results of the qualitative test were 11% points lower. cIg LC correctly identified 65% of BCL with dual positive sIg LC; 36% of BCL with difficult to interpret sIg LC and only 7% of BCL with negative sIg LC. The best results in differentiating between BCL and RLP were obtained when all three tests were used together. In samples with inconclusive sIg LC and additional monoclonal or polyclonal populations the κ:λ ratios did not differentiate between RLP and BCL. We propose that in case of inconclusive sIg LC Bcl-2 test is used first. The addition of cIg LC test is sensible only in cases with dual positive and difficult to interpret sIg LC.

## 1. Introduction

Flow cytometric immunophenotyping (FCI) has made a great contribution towards higher accuracy in lymphoma recognition from fine needle aspiration (FNA) biopsies over the past years. Several papers in the literature have reported from 80–99% correct diagnoses in differentiating between lymphoma and reactive lymphocytic proliferation (RLP) [1,2,3,4,5,6]. In order to make the distinction between B-cell lymphoma (BCL) and RLP cytopathologists rely on immunophenotypic characteristics which are more or less specific for some BCL and on the clonality of B-cells. However, clonality, which is usually determined on the basis of surface immunoglobulin light chain (sIg LC) ratio, cannot be determined in all cases. Therefore, additional analyses have to be applied, such as determination of Bcl-2 expression and the ratio between cytoplasmic immunoglobulin light chains (cIg LC). Bcl-2 is not overexpressed in most cases of RLP while overexpression has been shown immunohistochemically in tissue sections in 67–97% of BCL, depending on the specific BCL type [7,8]. The percentages of Bcl-2 expression in individual lymphoma types vary somewhat in different reports. Furthermore, clonality determined by cIg LC ratio has been shown to support a lymphoma diagnosis [9]. In our previous paper we have already reported some results on the outcome of Bcl-2 and cIg LC tests in lymphocytic proliferations with inconclusive sIg LC [10]. However, the number of investigated cases was low and Bcl-2 expression was determined only qualitatively. We are not aware of any other publications where diagnostic accuracy of Bcl-2 and cIg LC tests were systematically investigated on FNA biopsy material. In most studies Bcl-2 and cIg LC tests have been used only in individual, complicated cases. Only Laane et al. have reported more extensively on the outcome of Bcl-2 expression in 189 cases of BCL and 137 cases of RLP [11].

The aim of our study was to test the diagnostic value of two additional tests, using FCI in differentiating between BCL and RLP in FNA samples from lymph nodes, most of which had inconclusive sIg LC: firstly, the qualitative and quantitative Bcl-2 tests were used; Secondly the clonality determination was conducted by cIg LC ratio alone, and in combination with the Bcl-2 test.

## 2. Results

### 2.1. Bcl-2 Expression

We determined Bcl-2 expression in 315 FNA samples from lymph nodes of 282 patients. There were 143 males and 139 females. The age of patients ranged from four to 92 years. Final diagnoses were RLP in 159 cases and BCL in 156 cases. There were 118 primary and 38 secondary lymphomas. Among BCL there were 68 follicular lymphomas (FL), 42 diffuse large B-cell lymphomas (DLBCL), 37 marginal zone lymphomas (MZL), four chronic lymphatic leukaemia (CLL), three Burkitt lymphomas (BL), one mantel cell lymphoma (MCL) and one unclassified lymphoma. Histological diagnoses were available for 187 patients.

FCI test defined 19 samples with monotypic sIg LC expression, 16 were polytypic and 280 samples had inconclusive sIg LC (208 negative, 54 dual positive and 18 difficult to interpret). The percentage of cells with negative sIg LC ranged from 17% to 100% (median = 60%), percentage of cells with dual positive sIg LC ranged from 7% to 100% (median 32%). The monotypic group contained only BCL, the polytypic group contained 15 RLP and one BCL. Among samples with inconclusive sIg LC the dual positive sIg LC group contained the highest number of BCL (49/54; 91%), followed by the “difficult to interpret group” (13/18; 72%). The negative sIg LC group contained only 36% BCL (74/208).

#### 2.1.1. Qualitative Bcl-2 Test

When we determined Bcl-2 expression qualitatively 32% of samples (102/315) showed overexpression. The test was correctly positive in 101/156 (65%) and falsely negative in 55/156 (35%) samples of BCL. The 55 false negative samples originated from 20/42 DLBC, 16/37 MZL, 14/68 FL, 3/3 BL and 2/4 CLL. There was one false positive Bcl-2 test. It was a sample from a patient with cytological diagnosis of CLL, who experienced complete regression of enlarged lymph nodes. Except for the cytologically diagnosed CLL with spontaneous regression, none of the samples from RLP showed Bcl-2 overexpression.

#### 2.1.2. Quantitative Bcl-2 Test

In quantitative determination of Bcl-2 expression the median value of Bcl-2 index was 1.0 in RLP (range 0.5–4.7) while the median value in BCL was 2.19 (range 0.6–13.0). The highest value of Bcl-2 index among RLP was in the sample from the patient with cytological diagnosis of CLL with spontaneous regression of enlarged lymph nodes. After we excluded this sample, the highest value was 1.5.

**Figure 1:**
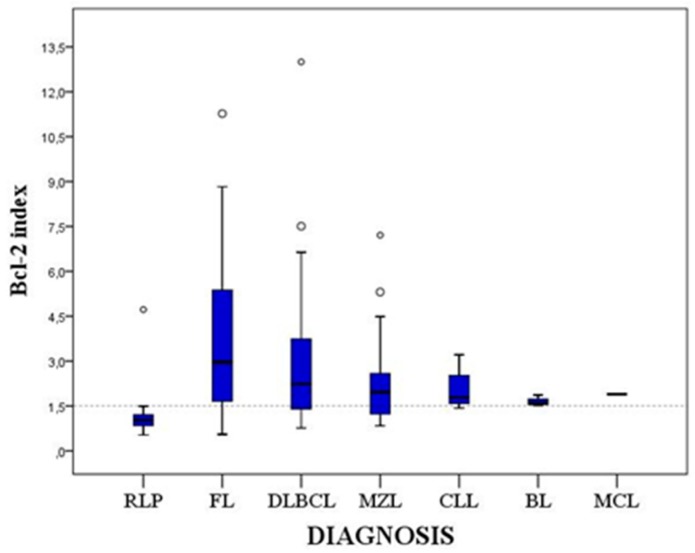
Quartile diagram of Bcl-2 indexes in RLP and various BCL types. The Bcl-2 index of 1.5 is shown with a dotted line. RLP—reactive lymphocytic proliferation. FL—follicular lymphoma. DLBC—diffuse large B-cell lymphoma. MZL—mantle zone lymphoma. CLL—chronic lymphatic leukaemia. BL—Burkitt lymphoma. MCL—mantel cell lymphoma.

Since area under curve (AUC) for Bcl-2 index values was 0.89 (95% confidence interval: 0.85–0.93, *p* < 0.001) we considered Bcl-2 test as excellent for differentiating between RLP and BCL. We obtained the best combination of sensitivity and specificity at Bcl-2 index value of 1.5 for differentiating between RLP and BCL (sensitivity 75%, specificity 99%). The positive quantitative Bcl-2 test (Bcl-2 index › 1.5) had a high positive prediction value (99%) and somewhat lower negative predictive value (80%). Bcl-2 indexes were statistically significantly higher (*p* < 0.001) in BCL compared to RLP. The same was true for the following specific lymphoma types: FL, DLBCL and MZL. CLL, BL and MCL also showed higher Bcl-2 indexes compared to those in RLP. However, they were represented in too few numbers for conclusive statistical analysis (Figure 1).

Quantitative Bcl-2 test was correctly positive in 118/156 (76%) cases, falsely negative in 38/156 (24%) cases and falsely positive in one case, the same one as explained for the falsely positive qualitative Bcl-2 test. Among the false negative cases, there were 13/42 DLBCL, 11/68 FL, 13/37 MZL and 1/4 CLL. Except for the cytologically diagnosed CLL with spontaneous regression, none of the RLP cases showed Bcl-2 overexpression. Therefore, quantitative Bcl-2 test correctly identified 88% of all cases, among them 76% of BCL.

Among samples with inconclusive sIg LC the Bcl-2 index was most frequently positive in the dual positive sIg LC group (84%; 41/49 BCL), followed by the “difficult to interpret” sIg LC group (77%; 10/13 BCL) and the negative sIg LC group (70%; 52/74 BCL). In the group with monotypic sIg LC, Bcl-2 test was positive in 79% (15/19 BCL). In the group with polytypic sIg LC one case showed positive Bcl-2 test (1/16).

#### 2.1.3. Comparison Between Qualitative and Quantitative Bcl-2 Tests

**Figure 2:**
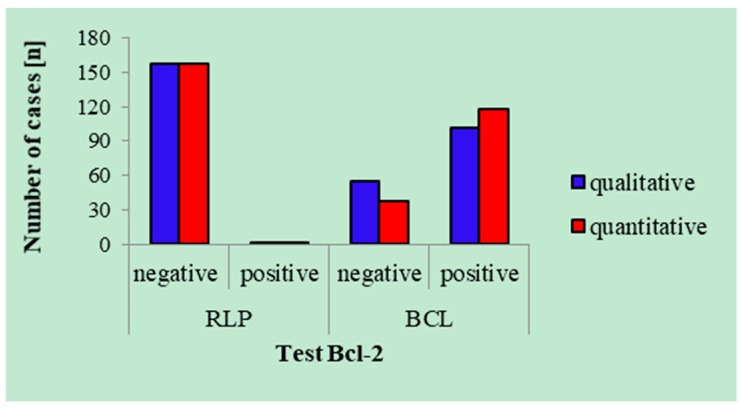
Results of the qualitative and quantitative Bcl-2 tests (Bcl-2 index of 1.5). RLP—reactive lymphocytic proliferation. BCL—B-cell lymphoma.

Results of the qualitative and quantitative Bcl-2 tests were the same in all cases of RLP and in the case of CLL with spontaneous regression of lymph nodes. The two tests were not compatible in 20 cases (16%) of BCL (Figure 2).

**Figure 3:**
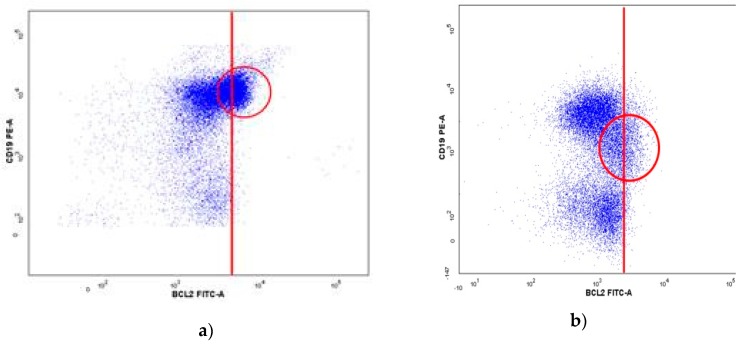
Positive qualitative Bcl-2 test in two samples with negative quantitative Bcl-2 test. Results of Bcl-2 measurement in B-cell and in T-cells, where we observe two populations of B-cells with different expression of Bcl-2 and a population of T-cells. (**a**) a small B-cell population with Bcl-2 overexpression which was not detected by the quantitative test. (**b**) Positive qualitative Bcl-2 test with few T-cells which resulted into false negative quantitative Bcl-2 test. Red circles show B-cells with Bcl-2 overexpression.

In 18 samples only the quantitative test was positive while in two samples only the qualitative test was positive. The latter two samples contained many reactive B-cells in addition to the neoplastic ones. In one of these two samples, the quantitative Bcl-2 test was negative because there were too few B-cells with Bcl-2 overexpression, while in the other sample the quantitative test was negative because there were too few T cells (Figure 3a,b). In 18 samples the qualitative test was negative because the difference in median expression of Bcl-2 between B-cells and T cells was too low. Among these 18 samples there were 8 DLBCL, 4 FL, 3 MCL and 3 BL.

Sensitivity, specificity, PPV and NPV for each type of Bcl-2 test as well as for the combination of both types are shown in Table 1.

### 2.2. Expression of cIg LC and Bcl-2

We determined expression of cIg LC and Bcl-2 in 232 FNA samples from lymph nodes of 211 patients. There were 106 males and 105 females. The age of patients ranged from 14 to 92 years. Final diagnoses were RLP in 102 cases and BCL in 130 cases, among them 100 primary and 30 secondary lymphomas. Histology was available for 153 patients.

In this study group, five samples had monotypic sIg LC, seven were polytypic and 220 had inconclusive sIg LC. The percentage of cells with negative and dual positive sIg LC was the same as in the study group where only Bcl-2 testing was performed. Among 130 BCL, five samples expressed monotypic sIg LC, one expressed polytypic sLC and 124 sIg LC were inconclusive (70 negative, 43 dual positive and 11 difficult to interpret). Among 102 RLP, there were no samples with monotypic sIg LC, six expressed polytypic sIg LC and 96 expressed inconclusive sIg LC (89 negative, four dual positive and three difficult to interpret).

In 5% of all cases both sIg and cIg LC were monotypic or polytypic. 20% of samples had inconclusive sIg LC and conclusive cIg LC. 75% of samples had inconclusive both sIg and cIg LC. Expression of cIg LC in groups with specific expression of sIg LC is shown in Table 2. With the use of cIg LC test we identified 7% (8/70) of BCL within the group of samples with negative sIg LC, 65% (28/43) of BCL within the group of dual positive sIg LC and 36% (4/11) of BCL within the group of sIg LC which were difficult to interpret. There was one BCL among samples with inconclusive sIg LC and polytypic cIg LC.

### 2.3. Usefulness of Bcl-2 Test and Determination of cIg LC in Samples with Inconclusive sIg LC

**Figure 4:**
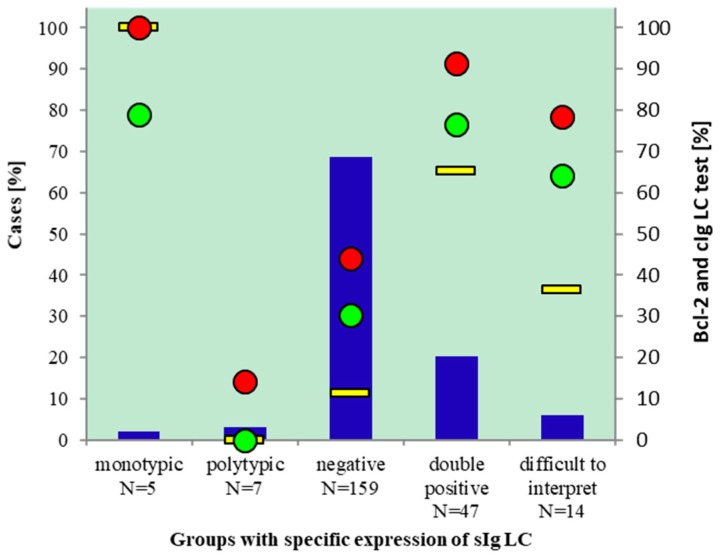
Performance of Bcl-2 and cIg LC tests for diagnosing BCL in cases with inconslusive sIg LC. Samples with conclusive and inconclusive sIg LC (blue columns, left axis), % BCL (red dots, right axis), % monotipic cIg LC (yellow line, right axis), % positive Bcl-2 test (green dots, right axis).

In Table 3 we present sensitivity, specificity, PPV and NPV of Bcl-2 test (qualitative and quantitative); of the cIg LC test; and of the combination of both tests together. For all three groups of inconclusive sIg LC sensitivity and specificity were highest when we performed both tests together. In the group with negative sIg LC, the sensitivity of Bcl-2 test was only 1.5 percentage points lower than the sensitivity for the combination of Bcl-2 and cIg LC tests together, while specificity stayed the same. In the other two groups of samples with inconclusive sIg LC there was nine percentage points difference in sensitivity. Determination of cIg LC is the least sensitive test for differentiation between RLP and BCL in samples with inconclusive sIg LC (Table 3, Figure 4).

### 2.4. Expression of Additional Monotypic or Polytypic Cell Populations in Samples with Inconclusive sIg LC

In the both above mentioned study groups we detected additional monotypic or polytypic B-cell populations among those with inconclusive sIg LC. The Bcl-2 test group contained 180/280 (64%) such cases, 160 with additional polytypic sIg LC populations and 20 with additional monotypic sIg LC. Samples with additional polytypic populations originated from 112 RLP and 48 BCL. Samples with additional monotypic populations originated from six BCL and one RLP. The cIg LC study group contained 129/220 samples with inconclusive sIg LC and additional monotypic (18/129) or polytypic (111/129) sIg LC populations. Samples with additional polytypic populations originated from 68 RLP and 43 BCL. Samples with additional monotypic populations originated from 17 BCL and one RLP.

We tried to differentiate between BCL and RLP on the basis of ratios between sIg LC of these additional populations by the use of ROC curve. However, the AUC for κ/λ fractions was too small to enable such a differentiation. The differentiation by κ:λ ratios of additional monotypic/polytypic populations was unsuccessful in both study groups.

## 3. Discussion

The results of our study demonstrated that qualitative Bcl-2 test correctly identified 82% of cases as BCL or RLP, while the quantitative Bcl-2 test correctly identified 88% of all cases when we used Bcl-2 index of 1.5 as the cut-off value. In both tests types, there was one, the same, falsely positive case. Only 21% of specimens with inconclusive sIg LC were correctly identified as BCL or RLP when clonality was determined by the cIg LC test. The best results were obtained when all three tests were applied together. Depending on the specific subgroup of samples with inconclusive sIg LC, the sensitivity ranged from 78% to 91% and the specificity from 99% to 100%.

Although we were primarily interested in differentiating between BCL and RLP with inconclusive sIg LC, we also included in our study 57 cases with conclusive sIg LC for which cytomorphology was not concordant with FCI outcome (35 cases in the group where only Bcl-2 was determined and 12 cases where Bcl-2 and cIg LC were determined). These cases also served as a control group. The results showed that the percentage of correctly positive Bcl-2 test determined quantitatively was almost the same in both groups: 76% in the group with inconclusive sIg LC and 79% in the group with monoclonal sIg LC. Among the 16 cases with polytypic sIg LC there was one case of BCL (DLBCL), however, it did not show Bcl-2 overexpression. Lymphomas with polytypic sIg LC have been observed previously. Laane et al., for example, reported 9/222 BCL with polytypic sIg LC [11].

Our results demonstrated a very high PPV of the Bcl-2 test. The only false positive case was the sample with morphologic characteristics of CLL and positive expression of CD5 and CD23. There was no histologic confirmation of the diagnosis since enlarged lymph nodes regressed spontaneously. However, the patient returned recently with enlarged lymph nodes, FNA was performed again and FCI demonstrated the same immunophenotype as ten years ago. The patient is still under diagnostic investigation. However, we believe that our diagnosis was correct and that the case was in reality not a false positive one regarding the Bcl-2 overexpression. There are reports in the literature of spontaneous regression of histologically confirmed lymphomas [12,13]. The diagnoses of some cases were supported by immunophenotyping, including Bcl-2 overexpression. Most of the reported cases, however, were aggressive lymphomas, predominantly of DLBC type. Abe et al. have reviewed the literature on spontaneous lymphoma regression and found one small cell lymphoma among 15 reported cases [13]. Iwatani et al. reported that many cases of spontaneous lymphoma regression involved invasive intervention such as core needle biopsy or excisional biopsy and that the duration of regression lasted for months or years [12]. Therefore, it is very likely that in our case spontaneous regression of CLL was the result of FNA and that longer follow up will prove the correct diagnosis.

We have found very few reports on Bcl-2 expression in BCL and RLP determined by FCI and none where these two tests would be investigated in samples with predominantly inconclusive sIg LC. Cornfield et al. investigated Bcl-2 expression on material from surgical biopsies and were concerned solely on the ability to distinguish between follicular hyperplasia and FL [14]. Cook et al. also used surgical biopsies and investigated the difference in Bcl-2 expression in 28 cases of RLP and 17 FL [15]. In addition, they determined Bcl-2 expression also in 20 various non-Hodgkin lymphomas and in Hodgkin lymphomas, with the maximum number of six cases per lymphoma type. We are aware of only two reports where Bcl-2 expression was investigated on FNA material and results reported in some detail [11,16]. Tarafder et al. investigated 10 cases of DLBC lymphomas for a variety of cell markers including Bcl-2 [16], while Laane et al. determined Bcl-2 expression on a large number of various samples including 189 BCL and 137 RLP [11].

Additional differences among the above mentioned papers and our report are also in the use of qualitative or quantitative Bcl-2 determination and in the manner in which Bcl-2 expression was detected. Tarafder et al. [16] used only the qualitative method while Cornfield et al. [14] and Laane et al. [11] used only the quantitative method. Cook et al. [15] used both methods as it was presented in our study. For Bcl-2 analysis we used a three color combination of Bcl-2, CD19 and CD45, Cornfield et al. [14] used Bcl-2 and CD20, Cook et al. [15] used Bcl-2, CD20 and CD10 and Laane et al. [11] applied the combination of Bcl-2, CD19 and CD10. Cook et al. [15] argue that the addition of CD10 helps to identify a specific B-cell subset of interest, especially if the specimen contains many normal B-cells with strong Bcl-2 expression. Furthermore, the authors mention that CD20 is not uniform in neoplastic cells of FL and therefore CD 20 intensity alone is not a reliable marker of follicular centre cells. They detected four cases of lymphoma with small CD10+ populations and high expression of Bcl-2. However, detection of small populations of cells with high expression of Bcl-2 may also be misleading. Laane et al. [11] reported 2/172 RLP cases with a subpopulation of cells showing high expression of Bcl-2, one was CD10+, the other was dim CD5+.

In the report of Cook et al. [15] the qualitative method of determining Bcl-2 overexpression proved very efficient for FL but was less successful for RLP and other lymphoma types. The qualitative method correctly identified 63% of all cases while we were able to correctly identify 82% of all case. The results of the quantitative Bcl-2 determination were more similar in the two studies. Using different approaches, we obtained the same value of 1.5 for the Bcl-2 index which best differentiated between BCL and RLP. In our study the quantitative Bcl-2 test was successful in 88% of all cases while in the study of Cook et al. [15] the test was successful in 82%. There is one interesting difference between our study and the study of Cook et al. [15]. In their study the qualitative Bcl-2 test performed equally well or worse compared to the quantitative test. In our study, the qualitative Bcl-2 test also identified less BCL than the quantitative test, however, it did identified two cases of BCL that were missed by the quantitative test.

Despite similarities between our study and the one reported by Laane et al. [11] in the type of material used and in the number of various entities investigated, we can compare the results of the two studies only to a limited extent since they are presented in different fashions. Laane et al. [11] did not attempt to differentiate between BCL and RLP on the basis of a cut-off point of Bcl-2 index. They measured the MFI values for Bcl-2 in malignant cells of various types of BCL as well as in B-cells of RLP and found the same results as we did: B-cells in RLP showed statistically significantly lower levels of Bcl-2 expression than did B-cells in BCL.

The Bcl-2 test has a very high PPV and somewhat lower NPV which varies in different BCL types and is the main drawback of the test. The major weakness of our study is that we could not calculate reliably the percentage of Bcl-2 negative individual BCL types because some of them were represented in very few numbers. For example, only four CLL were included in the study because they were mostly monotypic with a characteristic immunophenotype and additional FCI analyses were not necessary. FL, DLBC and MZL, on the other hand, were represented in moderately high numbers and in these three groups of BCL Bcl-2 was overexpressed in 84%, 69% and 65% respectively. These results differ only by one to two percentage points from the results reported by Lai et al. [7].

In our study group where we determined cIg LC in addition to the Bcl-2 expression 79% of samples with inconclusive sIg LC also expressed inconclusive cIg LC. Only 18% of samples with inconclusive sIg LC had monotypic cIg LC and they all originated from BCL. Three percent of samples had inconclusive sIg LC and polytypic cIg LC, however, one of them was a DLBCL. The Bcl-2 was not overexpressed in this case.

The reasons for inconclusive Ig LC have not been definitely explained. Some speculate that dual positive expression of LC is the result of unspecific binding of free immunoglobulins from serum and from tissues to the Fc receptors on lymphatic cells [17,18]. According to another theory, the aberrant LC expression can be the result of altered genes for LC expression [19,20,21,22]. Our results support both theories. In the group with dual positive sIg LC we detected 63% of cases with monotypic or polytypic cIg LC. These results could be partly explained by unspecific binding of free Ig during the preparation for sIg LC detection since this protocol includes fewer washes compared to the protocol for the preparation of surface antigens. Britt et al. demonstrated that there was a significant benefit in determining the κ/λ ratio from one to two washes and from two to three washes but not further [23]. The 16% of cases with dual positive both sIg and cIg LC would support the altered gene expression theory. This theory can further be supported by our results of negative sIg LC where most cases did not express the cIg LC.

We have found few articles which report on the cIg LC [9,24,25,26]. Most of these reports discuss cIg LC in the diagnosis of CLL because sIg LC are frequently negative or weakly expressed in this lymphoma type. Lewis et al.l found cases of CLL which did not express either sIg LC or the characteristic immunophenotype [9]. With detection of cIg LC they were able to demonstrate monoclonality in 6/7 cases of CLL. Similar results were reported also by Bardales et al. [24] and by Coser et al. [26], who demonstrated monotypic cIg LC in 6/9 and in 9/10 cases of CLL, respectively. Babušikova et al., on the other hand, argued that determination of cIg LC in CLL is not necessary since most cases of CLL express the characteristic immunophenotype [25]. For the same reason our study included only four cases of CLL. The expression of cIg LC was helpful only in one case with monotypic cIg LC while cIg LC were negative in two cases and polytypic in one case. Therefore we also believe that demonstration of cIg LC is not very helpful in CLL. However, according to our results, it can be helpful in other BCL types with inconclusive sIg LC. We demonstrated monotypic cIg LC in 10/35 DLBCL and in 15/56 FL.

Whenever we fail to differentiate between BCL and RLP by using morphology and FC, including the analysis of BCL-2 and cIg LC, it is advisable to use molecular techniques. In our previous paper we have already reported that we were able to demonstrate monoclonality in 77% of BCL by detecting immunoglobulin heavy chain rearrangement using PCR [10]. However, results should be used with caution, since we observed that monoclonality was present also in 12% of our RLP cases.

## 4. Materials and Methods

We included FNA samples of lymph nodes obtained between the years 2007 and 2013 for which Bcl-2 and cIg LC were determined in addition to routinely performed FCI antibody panels. Most of these samples had inconclusive sIg LC, however, all were not difficult cases for interpretation since many had characteristic morphology and/or immunophenotype. In addition, we included some cases with conclusive sIg LC because their cytomorphology was not concordant with FCI outcome. From the hospital information system we obtained clinical data, cytological and histological diagnoses for all patients included in the study. The study was approved (February 18th, 2014) by The National Medical Ethics Committee of the Republic of Slovenia (109/02/14) and was performed in compliance with the Helsinki declaration.

Histological diagnosis was considered final and was based on microscopic examination and on the results of immunohistochemistry. For patients without histological diagnosis we determined final diagnosis on the basis of cytological diagnosis and data on clinical course of the disease. Cytological diagnoses of RLP or BCL were based on morphological criteria of lymphoid cells combined with the results of FCI analysis, namely the expression of CD45, CD20, CD19, CD3, FMC7, CD10, CD5, CD23, sIg LC, cIg LC and Bcl-2. The average follow-up period was 4.8 years.

The preparation of cell suspension from FNA lymph node sample, cell counting, the sample preparations for FCI (including preparation for determination of cIg LC and Bcl-2), acquisition of cells with flow cytometer and measurement result analysis have already been described in our previous paper [10].

The Ig light chains (Ig LC) were considered conclusive when B-cell populations were present only within κ or/and λ positive area on FCI histogram. The Ig LC were considered inconclusive when B-cells were in the negative or dual positive area of the histogram or when we could not definitively determine the ratio between Ig LC. The later situation presented in cases with more than two populations of cells expressing κ or λ LC and in cases with few CD19 positive cells. This group of inconclusive Ig LC was named “difficult to interpret". Among samples with inconclusive sIg LC there were many cases with additional monotypic or polytypic B-cell populations. In such samples we investigated the possibility to differentiate between BCL and RLP on the basis of the ratio between sIg LC of these additional populations.

We determined the expression of Bcl-2 qualitatively and quantitatively. Qualitative method represented visual determination of FCI results. The method was already explained in our previous paper. We determined Bcl-2 quantitatively by calculating the Bcl-2 index, which is the ratio between mean fluorescence intensity (MFI) of B-cells and MFI of T-cells T within the same sample.

For statistical analysis of results we used the programme Statistical Package for Social Sciences (IBM Corporation, New York, NY, USA). We used the curve of receiver operating characteristics (ROC) for analyzing the following results: 1. For determining cut-off point values for Bcl-2 index, which enables most accurate differentiation between RLP and BCL; 2. For determining if it is possible to differentiate between RLP and BCL on the basis of sIg LC ratio in samples with inconclusive sIg LC and expressing additional polytypic or monotypic lymphoid cell populations; 3. By comparing the sIg LC and cIg LC tests we investigated whether additional analysis of cIg LC increases recognition between RLP and BCL.

ROC curve demonstrates the ratio between fractions of true positive and false positive diagnoses at various values of Ig LC ratios or various values of Bcl-2 index. To produce an ROC curve we set the number of samples with true positive diagnosis (sensitivity-y coordinate) and the number of samples with false positive diagnosis (1-specificity –x coordinate) for each value of the Ig LC ratio or Bcl-2 index. Then we selected the cut-off value of the Ig LC ratio or Bcl-2 index at which we obtained the highest sensitivity at maximum specificity for differentiating between RLP and BCL. We constructed separate ROC curves for all RLP and BCL with predominant expression of κ sIg LC and separate curves for RLP and BCL which predominantly expressed λ sIg LCs.

By calculating the area under the ROC curve we determined the probability of a certain test to differentiate between RLP and BCL. The larger the AUC the more successful is the test. Values of AUC range from 0 to 1. The usefulness of the test is divided into five categories: unsatisfactory (AUC = 0.5), poor (0.5 < AUC <0.7), acceptable (0.7 ≤ AUC < 0.8), excellent (0.8 ≤ AUC < 0.9), and outstanding (AUC ≥ 0.9) [27].

## 5. Conclusions

On the basis of our results we propose the following algorithm for the use of Bcl-2 and cIg LC in samples with inconclusive sIg LC. The Bcl-2 test should be used first. In the case that sIg LC are negative, the addition of cIg LC is not recommended since the sensitivity of Bcl-2 and cIg LC tests together is only 1.5 percentage points higher compared to the use of Bcl-2 test alone. The use of both tests together is advisable only in the groups of dual positive and difficult to interpret sIg LC where the sensitivity is nine percentage points higher compared to the sensitivity of the Bcl-2 test alone.

## Figures and Tables

**Table 1:** Sensitivity, specificity, PPV and NPV of the qualitative and quantitative Bcl-2 test for differentiation between RLP and BCL.

Test Bcl-2	Sensitivity [%]	Specificity [%]	PPV [%]	NPV [%]
Qualitative	65	99	99	74
Quantitative	75	99	99	80
Both tests together	76	99	99	81

PPV—positive predictive value. NPV—negative predictive value.

**Table 2:** Expression of cIg LC in groups with specific expression of sIg LC in FNA of lymph nodes.

	sIg LC					
cIg LC	Negative N (L)	Double Positive N (L)	Difficult to Interpret N (L)	Monotypic N (L)	Polytypic N (L)	Total N (L)
Negative	145 (61)	7 (5)	1 (1)	-	-	153 (69)
Double positive	-	8 (7)	-	-	-	8 (7)
Difficult to interpret	1 (0)	3 (3)	9 (6)	-	-	13 (9)
Monotypic	8 (8)	28 (28)	4 (4)	5 (5)	-	45 (45)
Polytypic	5 (1)	1 (0)	-	-	7 (1)	13 (2)
Total	159 (70)	47 (43)	14 (11)	5 (5)	7 (1)	232 (130)

sIg LC—surface immunoglobulin light chains. cIg LC—cytoplasmic immunoglobulin light chains. N—number of cases. L—number of B-cell lymphomas.

**Table 3:** Sensitivity, specificity, PPV and NPV of the Bcl-2 test (qualitative and quantitative), of the cIg LC test and of the two tests together in groups with inconclusive sIg LC.

Groups with Inconclusive sIg LC	Test	Sensitivity [%]	Specificity [%]	PPV [%]	NPV [%]
**Negative**	Bcl-2	76.92	98.94	98.04	86.11
	cIg LC	5.19	80.00	88.89	2.67
	Bcl-2 and/or cIg LC	78.46	98.94	98.08	86.92
**Dual positive**	Bcl-2	86.05	100.00	100.00	40.00
	cIg LC	65.22	100.00	100.00	5.88
	Bcl-2 and/or cIg LC	95.35	100.00	100.00	66.67
**Difficult interpretation**	Bcl-2	81.82	100.00	100.00	60.00
	cIg LC	28.57	-	100.00	-
	Bcl-2 and/or cIg LC	90.91	100.00	100.00	75.00

PPV—positive predictive value. NPV—negative predictive value. sIg LC—surface immunoglobulin light chains. cIg LC—cytoplasmic immunoglobulin light chains. The symbol ‘-’ indicates no true negative or false positive results.

## Supplementary Materials

Supplementary File 1

## Abbreviations

BCL	B-cell lymphoma
RLP	reactive lymphocytic proliferation
FNA	fine needle aspiration
sIg LC	surface immunoglobulin light chains
cIg LC	cytoplasmic immunoglobulin light chains
FCI	flow cytometric immunophenotyping
RLP	reactive lymphocytic proliferation
FL	follicular lymphoma
DLBCL	diffuse large B-cell lymphoma
MZL	marginal zone lymphoma
CLL	chronic lymphatic leukaemia
BL	Burkitt lymphoma
MCL	mantel cell lymphoma
AUC	area under curve
