# Supplementary material for: Usefulness of Bcl-2 Expression and the Expression of Cytoplasmic Immunoglobulin Light Chains in the Differentiation Between B-Cell Lymphoma and Reactive Lymphocytic Proliferations in FNA
Source: Int J Mol Sci. 2019 May 29;20(11):2648. doi: 10.3390/ijms20112648 (PMC6601031; doi:10.3390/ijms20112648)
Supplement: Supplementary File 1 [file ijms-20-02648-s001.pdf]

Table S1. TP, TN, FP and FN cases of the Bcl-2 test (qualitative and quantitative), of the cIg LC test and of the two tests together in groups with inconclusive sIg LC.

Groups with inconclusive sIg LC	Test	TP	TN	FP	FN
**Negative** (N=159)	Bcl-2	50	93	1	15
	cIg LC	8	4	1	146
	**Bcl-2 and/or cIg LC**	51	93	1	14
**Dual positive** (N=47)	Bcl-2	37	4	0	6
	cIg LC	30	1	0	16
	**Bcl-2 and/or cIg LC**	41	4	0	2
**Difficult interpretation** (N=14)	Bcl-2	9	3	0	2
	cIg LC	4	0	0	10
	**Bcl-2 and/or cIg LC**	10	3	0	1

sIg LC surface immunoglobulin light chains, cIg LC cytoplasmic immunoglobulin light chains, TP true positives, TN true negatives, FP false positives, FN false negatives, N number of cases
